# Beta-adrenergic agonism protects mitochondrial metabolism in the pancreatectomised rat heart

**DOI:** 10.1038/s41598-024-70335-4

**Published:** 2024-08-21

**Authors:** Ross T. Lindsay, Louise Thisted, Nora E. Zois, Sebastian T. Thrane, James A. West, Keld Fosgerau, Julian L. Griffin, Lisbeth N. Fink, Andrew J. Murray

**Affiliations:** 1 Department of Physiology, Development and Neuroscience, University of Cambridge, Cambridge, UK; 2 Gubra A/S, Hørsholm Kongevej 11B, 2970 Hørsholm, Denmark; 3 Department of Biochemistry and Systems Biology Centre, University of Cambridge, Cambridge, UK; 4 Present Address: Ascendis Pharma A/S, Hellerup, Denmark; 5 Present Address: AstraZeneca, Cambridge, UK; 6 Present Address: Pephexia Therapeutics ApS, Copenhagen, Denmark; 7 Present Address: The Rowett Institute, University of Aberdeen, Aberdeen, UK; 8 Present Address: Ferring Pharmaceuticals A/S, Kastrup, Denmark

**Keywords:** Mitochondria, Pancreatectomy, Adrenergic, Heart, Diabetes, Cardiomyopathy, Cardiomyopathies, Energy metabolism, Type 1 diabetes, Mitochondria, Cardiovascular biology, Metabolism

## Abstract

The diabetic heart is characterised by functional, morphological and metabolic alterations predisposing it to contractile failure. Chronic sympathetic activation is a feature of the pathogenesis of heart failure, however the type 1 diabetic heart shows desensitisation to β-adrenergic stimulation. Here, we sought to understand the impact of repeated isoprenaline-mediated β-stimulation upon cardiac mitochondrial respiratory capacity and substrate metabolism in the 90% pancreatectomy (Px) rat model of type 1 diabetes. We hypothesised these hearts would be relatively protected against the metabolic impact of stress-induced cardiomyopathy. We found that individually both Px and isoprenaline suppressed cardiac mitochondrial respiration, but that this was preserved in Px rats receiving isoprenaline. Px and isoprenaline had contrasting effects on cardiac substrate metabolism, with increased reliance upon cardiac fatty acid oxidation capacity and altered ketone metabolism in the hearts of Px rats, but enhanced capacity for glucose uptake and metabolism in isoprenaline-treated rats. Moreover, Px rats were protected against isoprenaline-induced mortality, whilst isoprenaline elevated cGMP and protected myocardial energetic status in Px rat hearts. Our work suggests that adrenergic stimulation may be protective in the type 1 diabetic heart, and underlines the importance of studying pathological features in combination when modeling complex disease in rodents.

## Introduction

Type 1 diabetes is associated with increased mortality in comparison with the general population, with cardiovascular diseases being the major cause of death in older patients^1^. Whilst hypertension and coronary artery disease are prevalent in these patients, the incidence of heart failure remains high even when these are accounted for. Of note, patients are susceptible to the development of diabetic cardiomyopathy (DbCM), a condition characterised by multiple structural, functional and metabolic alterations^2^. There remains a great need to better understand the link between DbCM and heart failure, and in particular how molecular derangements of the diabetic heart interact with other common components of cardiac pathology^3,4^.

The healthy heart is metabolically flexible, consuming a variety of metabolic substrates to meet ATP requirements depending on circumstances. Around 60–70% of myocardial ATP demand is met by fatty acid oxidation (FAO), but with an increased reliance on glucose metabolism during the insulin-stimulated, post-prandial state and in response to stresses such as exercise and hypoxia. Type 1 diabetes greatly increases the reliance of the heart on FAO, reducing the capacity to increase glucose oxidation in response to metabolic stress^5,6^. As such, isolated, perfused hearts from type 1 diabetic rats have low glucose oxidation capacities, even when measured in the absence of fatty acids^7^.

Alongside this impaired flexibility in substrate metabolism, cardiac mitochondrial alterations are typically observed in both animal models of type 1 diabetes and in patients^2^. For instance, in rats injected with streptozotocin (STZ) to ablate pancreatic beta-cell function, cardiac mitochondrial respiratory capacity is suppressed^8–10^. This was associated with an increased ratio of FAO to glucose oxidation capacity when measured in hearts ex vivo^8,11–14^, and with increased production of reactive oxygen species (ROS)^9,15^.

Impaired flexibility of myocardial substrate utilisation and mitochondrial impairments have been extensively associated with impaired contractile function in both patients with advanced heart failure and experimental rodent models of failure, including those of non-metabolic origin^16,17^. Chronic sympathetic nervous system activation is a prominent neurohormonal feature of the pathogenesis of heart failure, and the synthetic β-adrenoreceptor agonist, isoprenaline has been administered experimentally to rodents resulting in a stress-induced cardiomyopathy phenotype^18^. In rats, this promotes alterations in cardiac morphology, impaired contractile function and altered metabolism^19,20^, including a suppression of FAO^20^. β-adrenergic stimulation might therefore be expected to further worsen substrate metabolism and energetics in the diabetic heart; however type 1 diabetes is associated with decreased responsiveness to β-stimulation and lower densities of cardiac β-adrenoreceptors^21^.

In previous work, we superimposed isoprenaline administration onto a surgically induced rat model of type 1 diabetes (90% pancreatectomy; Px) to explore the impact on cardiac phenotype^4^. We found that Px alone was associated with myocardial remodelling, but elicited no difference in ejection fraction, whilst isoprenaline decreased ejection fraction and induced fibrosis^4^. There was no apparent interaction between the two interventions that worsened cardiac pathology, and whilst first exposure to isoprenaline resulted in 43% mortality in sham-operated animals, there were no deaths in the Px rats following isoprenaline treatment^4^. Of note, gene expression analysis indicated downregulation of the β2-adrenoreceptor (though not the β1-adrenoreceptor) in the hearts of Px rats, suggesting a possible mechanism for this protection^4^.

Here, we sought to understand the cardiac mitochondrial and metabolic consequences of 90% Px and isoprenaline, both alone and in combination, hypothesising that the hearts of Px rats would be relatively protected against the metabolic consequences of isoprenaline administration in comparison to those of non-diabetic, sham-operated rats.

## Research design and methods

### Animal experimentation

All work was approved by The Danish Animal Experiments Inspectorate (license no. 2019-15-0201-01648, 2017-15-0201-01183) and conformed to the European Parliament Directive on the Protection of Animals Used for Scientific Purposes (2010/63/EU). All experiments were carried out according to the relevant guidelines and regulations. This study is reported in accordance with the ARRIVE guidelines.

As reported previously^4^, male Sprague–Dawley rats (NTac:SD) (8–10 weeks old, 210–280 g, Taconic Biosciences) were randomised into groups undergoing either removal of 90% of the pancreas (pancreatectomy, Px) or a sham operation. Following an overnight fast, rats received subcutaneous injections of atropine (0.1 mg/kg), enrofloxacin (50 mg/kg), carprofen (50 mg/kg) and saline (20 ml/kg) prior to surgery before operation under isoflurane (2–3%) induced anaesthesia. Pancreatectomy (90%) was performed by ligation and then removal of the tail, body and part of the pancreatic head by gentle abrasion with a dental applicator. The remaining 10% of the pancreas comprised the portion between the duodenal loop and the pancreatic duct. All major blood vessels to the stomach, spleen and gut were left intact. Sham operation consisted of anaesthesia and ligation of the equivalent portion of the pancreas, before the ligature was removed to leave behind a fully functional pancreas. Rats received no food from one day before until 1 day after surgery, whereupon they were offered 5 g of chow. From the second day post-surgery normal rodent chow and water was provided ad libitum. Throughout, rats were housed in conventional cages with a normal 12 h/12 h light/dark photoperiod.

Commencing 5 weeks post-operation, pancreatectomised and sham operated animals were randomised into groups which underwent daily subcutaneous administration of either isoprenaline hydrochloride dissolved in saline (1 mg/kg), or vehicle (saline), for 10 days. This yielded four study groups: sham-operated rats administered vehicle (Sham/Vehicle), Px rats administered vehicle (Px/Vehicle), sham-operated rates administered isoprenaline (Sham/Iso), and pancreatectomised rats administered isoprenaline (Px/Iso). Blood glucose and bodyweight were monitored throughout. Tail vein blood samples were collected weekly following a 4 h fast. Samples were collected in heparinised glass capillary tubes, and suspended in glucose/lactate system solution buffer (EKF-diagnostics, Germany). Blood glucose was measured using a BIOSEN c-Line glucose meter (EKF-diagnostics, Germany).

At 10 weeks post-operation, rats were euthanised with rising concentrations of CO_2_, with death confirmed by cervical dislocation. Hearts were extracted, and the left ventricle sectioned before being snap frozen for further analysis (RNAseq and mass spectrometry) or transferred fresh into ice-cold biopsy preservation solution (BIOPS; 2.77 mM CaK_2_EGTA, 7.23 mM K_2_EGTA, 6.56 mM MgCl_2_.6H_2_O, 20 mM taurine, 15 mM phosphocreatine, 20 mM imidazole, 0.5 mM DTT, 50 mM 4-morpholineethanesulfonic hydrate, and 5.77 mM Na_2_ATP, pH 7.1.) for immediate analysis of mitochondrial respiratory function using high-resolution respirometry.

To ensure statistical independence, only one tissue sample from each rat was used for each experiment. Each tissue sample, therefore, was considered to be an experimental unit.

### High-resolution respirometry

A section of non-frozen left ventricle was dissected into bundles of 6–8 fibres, and permeabilised with gentle rocking for 20 min at 4 °C in BIOPS with 50 μg/ml saponin^22^. Fibre bundles were then washed (3 × 5 min, 4 °C) in respiration medium (MiR05: 0.5 mM EGTA, 3 mM MgCl_2_.6H_2_O, 60 mM K-lactobionate, 20 mM taurine, 10 mM KH_2_PO_4_, 20 mM HEPES, 110 mM sucrose, 1 g/l defatted BSA, pH 7.1).

Cardiac fibre bundles (2–3 mg) were immediately added to Oxygraph-O2k chambers (Oroboros Instruments, Innsbruck, Austria) containing 2 ml MiR05 at 37 °C. Respiratory capacities were assessed as described previously^22,23^. Malate (2 mM) and octanoyl carnitine (0.2 mM) were initially added to stimulate LEAK respiration (MOct_L_), followed by 5 mM ADP to stimulate oxidative phosphorylation (OXPHOS) supported by β-oxidation (MOct_*P*_). Next, pyruvate (20 mM) was added (MOctPyr_*P*_) followed by glutamate (10 mM) to assess the capacity for complex I-supported respiration (MOctPyrG_*P*_), and cytochrome *c* (10 µM) to assess outer mitochondrial membrane integrity. Succinate (10 mM) was subsequently added to additionally activate complex II (GMS_*P*_), followed by 0.5 µM rotenone to inhibit complex I (S_*P*_). The ratio of octanoyl carnitine to pyruvate-supported OXPHOS was calculated to indicate relative capacity for fatty acid oxidation (FAO)^22^. Oxidative coupling efficiency (OCE) was assessed as (MOct_*P*_ − MOct_*L*_)/MOct_*P*_.

### Mass spectrometry

Metabolites were extracted from frozen left ventricle using a Bligh–Dyer method as described previously^24^. Levels of glycolytic intermediates plus ATP and phosphocreatine (PCr) in the aqueous extract were then assessed using a BEH amide column chromatography method coupled to a Quantiva triple-quadrupole mass spectrometer (Thermo Scientific).

Fractions were reconstituted in an acetonitrile: 10 mM ammonium carbonate solution (7:3 v/v, 100 μl) containing a 10 μM mixture of internal standards (200 ml; phenylalanine d5, valine d8, leucine d10). The column used was a 1.7 μm BEH amide column (150 × 2.1 mm), coupled to a Vanquish UHPLC + series (Thermo Scientific, UK) LC system and a TSQ Quantiva Triple Quadrupole Mass Spectrometer (Thermo Scientific). The mobile phase was pumped at 600 μl min^-1^ with mobile phase A 0.1% ammonium carbonate solution, and mobile phase B acetonitrile. Mobile phase A was held at 20% for 1.5 min, linearly increased to 60% over the next 2.5 min, held at 60% for 1 min, before being decreased back to the initial conditions (20% mobile phase A) over 0.1 min. The total run time was 6 min. Nitrogen at 48 mTorr, 420 °C was used as a drying gas for solvent evaporation and the UPLC column was conditioned at 30 °C. The Quantiva utilised PosNeg switching at voltages of 3.5 and − 2.5 kV for ionisation.

### Gene expression

RNAseq was employed with the purpose of analysing the expression of pre-defined target genes associated with major pathways of substrate metabolism, namely fatty acid uptake/oxidation, glucose uptake/glycolysis, and ketolysis/ketogenesis. RNA was purified from homogenised tissue using the NucleoSpin RNA Plus kit (Macherey–Nagel GmbH). 500 ng purified RNA from each sample was used to generate a cDNA library using the NEBNext® Ultra™ II Directional RNA Library Prep Kit from Illumina (New England Biolabs). The cDNA library was then sequenced on a NextSeq 500 using NextSeq 500/550 High Output Kit V2 (Illumina). Sequencing was carried out for 75 cycles and 2 × 8 index cycles. The sequencing data was aligned to the rat genome obtained from the Ensembl database using the Spliced Transcripts Alignment to a Reference (STAR) software. Gene expression as RPKM is presented relative to the sham/vehicle group.

### Statistics

Statistical analysis was performed using a two-way ANOVA, with Isoprenaline treatment and Pancreatectomy as the variables. Significant differences were further analysed by a Tukey’s post-hoc honest significance difference test. Statistical significance was considered at p < 0.05.

### Ethics approval and consent to participate

All work was approved by The Danish Animal Experiments Inspectorate (license no. 2019-15-0201-01648, 2017-15-0201-01183) and conformed to the European Parliament Directive on the Protection of Animals Used for Scientific Purposes (2010/63/EU).

## Results

### Blood glucose, body weight and mortality

**Figure 1:**
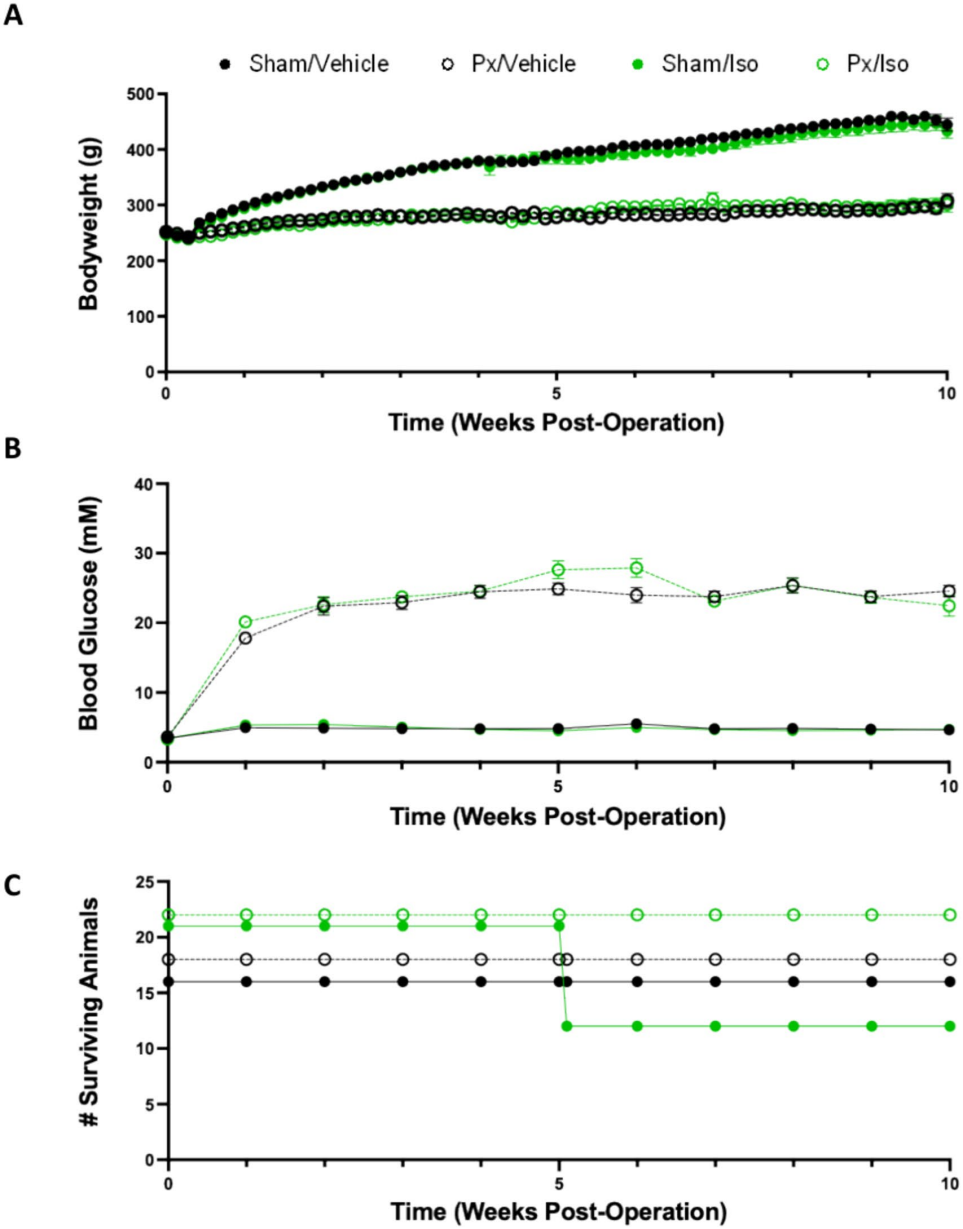
90% Pancreatectomy induced a diabetic phenotype, and protected against isoprenaline induced mortality. Sprague Dawley rats underwent 90% pancreatectomy (Px) or sham operation at week 0**.** Starting at 5 weeks, rats were administered once daily 1 mg/kg isoprenaline (Iso) or saline (vehicle) for 10 days. (**A**) Bodyweight (**B**) Fasted blood glucose and (**C**) Survival are charted over time following operation until termination of the study at 10 weeks. All displayed as mean ± SD, n = 12–18 for all groups in (**A**,**B**); n = 21–22 per group for (**C**).

Rats which underwent pancreatectomy gained less weight over the course of the 10 weeks than sham-operated counterparts (Fig. 1A), with final bodyweights for Px rats being 33% lower (p < 0.0001). Px rats reached hyperglycaemia within 1 week post-surgery, with blood glucose concentration reaching a plateau at 2 weeks post-surgery, of 22.4 ± 1.3 and 22.6 ± 1.16 mM for Px/Vehicle and Px/Iso rats, respectively (Fig. 1B). Sham-operated rats did not exhibit hyperglycaemia, with blood glucose concentrations of 4.9 ± 0.17 mM for Sham/Vehicle and 5.4 ± 0.25 mM for Sham/Iso. Neither blood glucose nor bodyweight were affected by isoprenaline administration.

Pancreatectomised rats were protected against the mortality induced by isoprenaline administration. Following receipt of the first administration of isoprenaline, 9 of the 21 sham-operated rats in the Sham/Iso group were humanely euthanised or died within the first 24 h following administration. In contrast, all pancreatectomised rats receiving isoprenaline survived, and as expected there was no mortality associated with vehicle administration (Fig. 1C).

### Cardiac mitochondrial respiratory capacity

Both pancreatectomy and isoprenaline administration suppressed aspects of cardiac mitochondrial respiratory capacity, although the nature of this suppression differed between the two interventions and there was a statistically significant interaction between the two.

**Figure 2:**
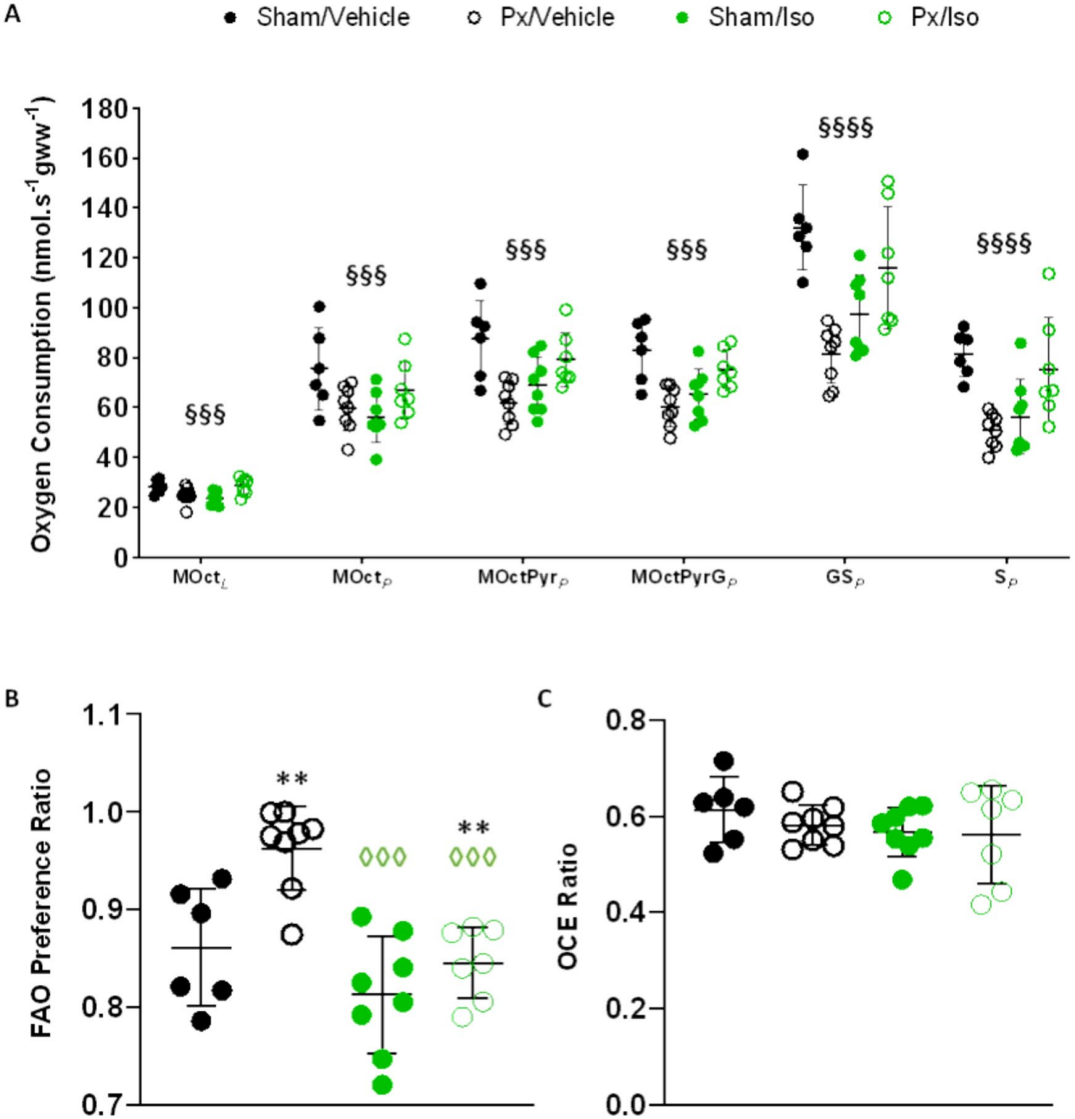
The combination of isoprenaline and pancreatectomy is mutually protective against mitochondrial injury, and restores metabolic flexibility impaired by Px. (**A**) Respiration rates corrected for wet mass. Subsequent additions were made of malate and octanoyl carnitine to initially stimulate leak respiration (MOct_L_), of ADP to stimulate β-oxidation supported Oxphos (MOct_*P*_), pyruvate for a comparison of substrate preference (MOctPyr_*P*_), glutamate to saturate complex I (MOctPyrG_*P*_), succinate to activate complex II (GS_*P*_*)*, and rotenone to inhibit complex I (S_*P*_). (**B**) The ratio between octanoyl carnitine and octanoyl carnitine plus pyruvate-supported respiration (FAO) as a marker of preference for fatty acid oxidation; (**C**) oxidative coupling efficiency ratio (OCE). All displayed as mean ± SD. 2-Way ANOVA **p < 0.01 effect of Px; ^◊◊◊^*p* < 0.001 effect of Iso; ^§§§^*p* < 0.001 and ^§§§§^*p* < 0.0001 interactions of Px and Iso. n = 7–10 for all groups.

Pancreatectomy alone, did not suppress LEAK or OXPHOS state respiration supported by octanoyl-carnitine (MOct_*L*_ or MOct_*P*_; Fig. 2A), suggesting a protection of fatty acid oxidation capacity. OXPHOS supported by complex I substrates was suppressed by pancreatectomy, with 28.9% lower respiration following the addition of pyruvate (MOctPyr_*p*_, p < 0.01) and 26.9% lower respiration following the addition of glutamate (MOctPyrG_*p*_, p < 0.01) in comparison with sham-operated rats. Maximal OXPHOS capacity supported by substrates for complex I and II in combination (GS_*P*_) was 38.4% lower in the hearts of pancreatectomised rats compared with sham-operated rats (p < 0.0001), whilst OXPHOS supported by the complex II substrate succinate (S_*P*_) was 37.7% lower (p < 0.01). Reflecting a relative protection of fatty acid oxidation in the hearts of pancreatectomised rats, the proportion of complex I activity that could be supported by FAO was 12% greater in the Px/vehicle group relative to sham/vehicle rats (Fig. 2B; Px effect, *p* < 0.01).

Isoprenaline administration resulted in a statistically significant suppression of mitochondrial respiratory capacity in all states measured (Fig. 2A). In the hearts of isoprenaline-treated rats, LEAK and OXPHOS respiration in the presence of malate and octanoyl-carnitine (MOct_*L*_ and MOct_*P*_) were 15.8% and 25.8% lower (p < 0.05) than in vehicle-treated rats. Complex I supported respiration rates were 20.1% and 20% lower for MOctPyr_*p*_ and MOctPyrG_*P*_, respectively (p < 0.05). Maximal OXPHOS capacity supported by substrates for complex I and II in combination (GS_*P*_) was 26.2% lower in the hearts of isoprenaline-treated rats compared with sham-operated rats (p < 0.01), whilst OXPHOS supported by the complex II substrate succinate (S_*P*_) was 30.9% lower in isoprenaline administered rats compared with sham-operated controls (p < 0.05). Isoprenaline treatment did not alter the proportion of complex I respiration that could be supported by FAO (Fig. 2B).

There was a statistically significant interaction between the effects of pancreatectomy and isoprenaline for all respiration rates measured (Px:Iso interaction effect p < 0.001 for all respiration rates, Fig. 2A). As such, the suppression of respiratory capacity seen in pancreatectomised rats compared with sham-operated controls was not seen in pancreatectomised rats treated with isoprenaline. OXPHOS respiration supported by complex I substrates was 27% higher (MOct_*p*_, p < 0.05) and 25% higher (MOctPyrG_*P*_, p < 0.05) in the hearts of Px/Iso rats compared with Px/Veh rats. Maximal OXPHOS capacity supported by substrates for complex I and II in combination (GS_*P*_) was 43% higher in the hearts of Px/Iso rats compared with Px/Veh rats (p < 0.01), whilst OXPHOS supported by the complex II substrate succinate (S_*P*_) was 48% higher (p < 0.05).

Neither pancreatectomy nor isoprenaline, alone or in combination, altered mitochondrial OXPHOS coupling efficiency (Fig. 2C).

Taken together, these results indicate that whilst pancreatectomy and isoprenaline independently suppressed components of cardiac mitochondrial respiratory capacity, when applied in combination respiratory capacity was preserved.

### Fatty acid oxidation genes

**Figure 3:**
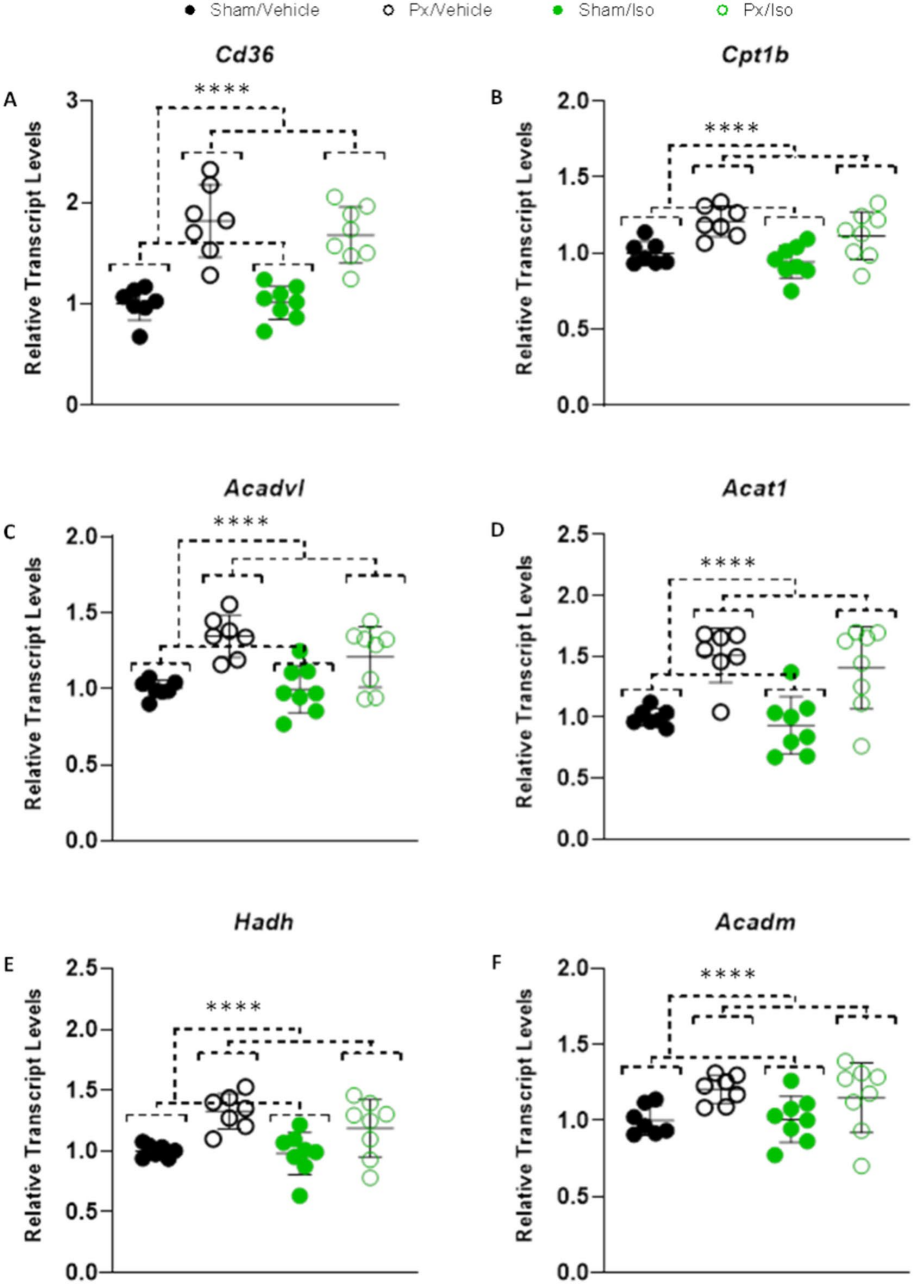
Greater expression of FAO related genes in Px hearts. Transcript levels of (**A**) *Cd36*, (**B**) *Cpt1b* (Carnitine Palmitoyl Transferase 1b), (**C**) *Acadvl* (Very long-chain specific acyl-CoA dehydrogenase), (**D**) *Acat* (Acyl-CoA:cholesterol acyltransferase), (**E**) *Hadh* (Hydroxyacyl-Coenzyme A dehydrogenase) and (**F**) *Acadm* (medium-chain acyl-CoA dehydrogenase) relative to Sham/Vehicle hearts, as measured by RNAseq. All displayed as mean ± SD. 2-way ANOVA ****effect of Px, *p* < 0.0001. n = 7–8 for all groups.

To further investigate the metabolic effects of pancreatectomy and isoprenaline administration, we measured the expression of genes encoding proteins that support myocardial fatty acid oxidation. Expression of *Cd36* (encoding the sarcolemmal fatty acid uptake transporter CD36/FAT) was 82% higher in the hearts of pancreatectomised rats compared with sham-operated controls (Px effect p < 0.0001; Fig. 3A). Similarly, cardiac expression of *Cpt1b* (encoding carnitine palmitoyl transferase 1, CPT1) was 21% higher (Px effect p < 0.0001) in pancreatectomised rats (Fig. 3b). Expression levels of *Acadvl*, *Hadh*, *Acat* and *Acadm* (encoding β-oxidation enzymes) were respectively 35%, 33%, 51% and 21% higher (Px effect p < 0.0001) in the hearts of pancreatectomised rats than in those of sham-operated rats (Fig. 3C–F). There was no significant effect of isoprenaline administration on the expression of any of these genes.

In conjunction with our respirometry results, these findings therefore suggest a relative preservation of FAO capacity in the hearts of pancreatectomised rats.

### Glucose metabolism

**Figure 4:**
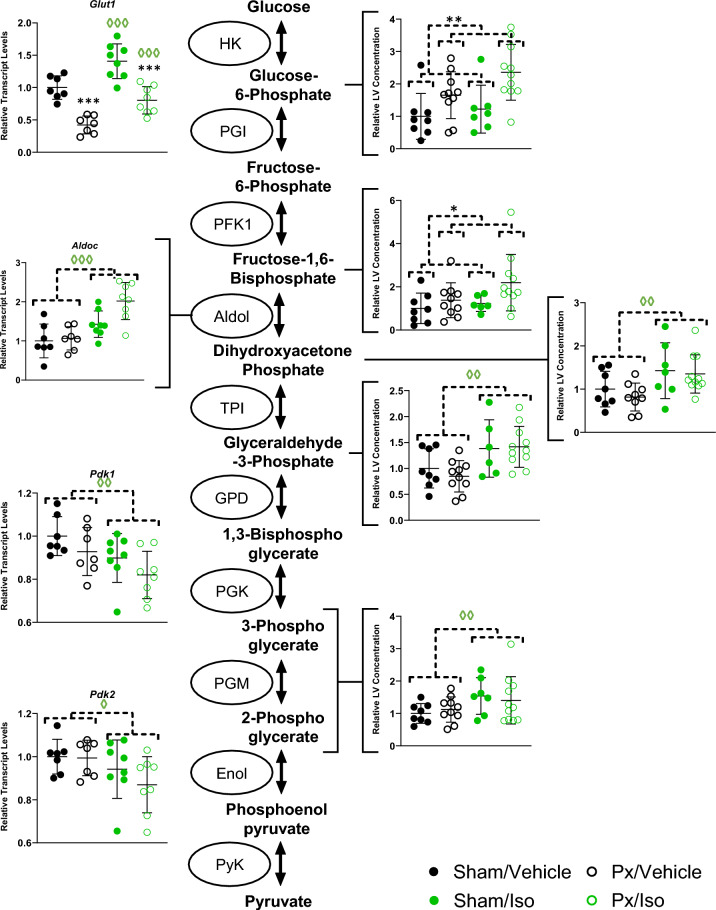
Isoprenaline administration leads to greater flux through glycolysis. Cardiac concentrations of glycolytic intermediates measured by LC–MS/MS (Right) n = 7–11 for all groups, and transcript levels of selected enzymes involved in glucose metabolism as measured by RNAseq (Left), All results displayed as mean ± SD. 2-way ANOVA *p < 0.05, **p < 0.01 and ***p < 0.001 effects of Px; ^◊^*p* < 0.05, ^◊◊^p < 0.01 and ^◊◊◊^*p* < 0.001 effect of Iso. n = 7–8 for all groups.

To further probe the effects of pancreatectomy and isoprenaline administration on cardiac substrate metabolism we measured the levels of glycolytic intermediates, and the expression of genes associated with glucose uptake and metabolism (Fig. 4).

Expression of *Glut1* (encoding the constitutive glucose uptake transporter GLUT1) was suppressed by pancreatectomy (Px effect p < 0.001) but enhanced by isoprenaline (Iso effect p < 0.001), such that expression levels were 58% lower in pancreatectomised rats compared with sham-operated controls, but 41% higher in isoprenaline-treated rats compared with vehicle-treatment.

Myocardial concentrations of early glycolytic intermediates were elevated in pancreatectomised rats compared with sham-operated controls (Px effect p < 0.01), with 65% higher levels of glucose-6-phosphate and 38% higher levels of fructose bisphosphate detected in these hearts. There was no significant effect of isoprenaline on these intermediates.

Myocardial expression of *Aldoc* (encoding aldolase C) was significantly enhanced by isoprenaline administration (Iso effect p < 0.001) with expression following isoprenaline treatment being 43% higher in the hearts of sham-operated animals and 100% higher in pancreatectomised rats. Furthermore, isoprenaline treatment significantly enhanced myocardial concentrations of glycolytic intermediates downstream of aldolase (Iso effect p < 0.01), with levels of dihydroxyacetone, glyceraldehyde-3-phosphate and 2/3-phosphoglycerate being 42%, 38% and 54% higher, respectively, in the hearts of isoprenaline-treated sham-operated rats, compared with vehicle-treated controls. Similarly, concentrations of the same three metabolites were respectively 35%, 42% and 40% higher in pancreatectomised rats following isoprenaline treatment, compared with vehicle-treated counterparts.

Isoprenaline administration also resulted in lower expression of genes encoding two pyruvate dehydrogenase kinase isoforms (*Pdk1* and *Pdk2*; Iso effect *p* < 0.01 and *p* < 0.05, respectively), potentially supporting greater rates of pyruvate flux through pyruvate dehydrogenase. Myocardial *Pdk1* expression was respectively 10% and 18% lower following isoprenaline administration in sham-operated and pancreatectomised rats respectively, whilst *Pdk2* expression was 6% and 14% lower in the same hearts.

To investigate metabolite levels in pathways into which glycolytic intermediates might be diverted, levels of pentose phosphate pathway metabolites, and of lactate, were also measured by MS. 6-phosphoglycerate levels were 31.4% higher in Px/Vehicle rats vs Sham/Vehicle controls, and 4.03-fold greater in LV from Px/Iso rats than in Sham/Iso LV (Supplementary Fig. 1A, Px effect, *p* < 0.05). There was no effect of either treatment upon ribulose-5-phosphate levels (Supplementary Fig. 1B), but ribose-5-phosphate levels were 40.5% higher in Sham/Iso rats vs Sham/Vehicle controls, and 63.1% higher in LV from Px/Iso rats than Px/Vehicle LV (Supplementary Fig. 1C, Iso effect, *p* < 0.05). Lactate levels were unchanged between the groups (Supplementary Fig. 1D).

These findings collectively point towards a greater capacity for glycolysis, and possible relative protection of pyruvate oxidation in the hearts of isoprenaline treated rats.

### Ketone body metabolism

**Figure 5:**
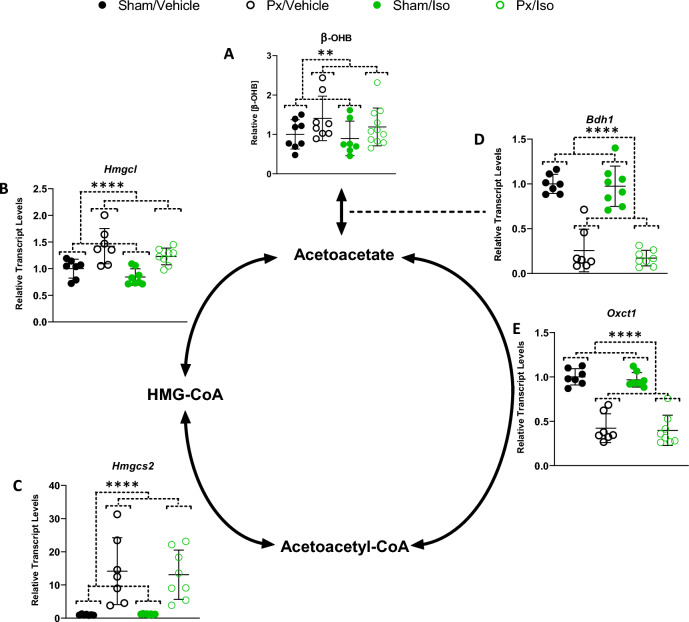
Altered Ketone Body Metabolism in the Diabetic Heart. (**A**) Left-ventricular β-hydroxybutyrate (β-OHB) concentration relative to sham/vehicle, as measured by LC–MS, n = 7–11 for all groups. Relative transcript levels of (**B**) *Hmgcl* (HMG-CoA Lyase), (**C**) *Hmgcs2* (mitochondrial HMG-CoA Synthase), (**D**) *Bdh1* (β-hydroxybutyrate Dehydrogenase), and (**E**) *Oxct1* (Succinyl-CoA Oxotransferase; SCOT) as measured by RNAseq. All results displayed as mean ± SD. 2-way ANOVA effects of Px: **p < 0.01, and ****p < 0.0001. n = 7–8 for all groups.

Next, we measured levels of the ketone body, β-hydroxybutyrate (β-OHB), and the expression of genes encoding enzymes that support myocardial ketone metabolism. Pancreatectomy resulted in elevated myocardial β-hydroxybutyrate (β-OHB) levels (Fig. 5A; Px effect *p* < 0.01), which were 41% greater in the hearts of pancreatectomised rats administered vehicle and 19% greater in those treated with isoprenaline, compared with the respective sham-operated controls. Expression of both *Hmgcl* (encoding HMG-CoA Lyase) and *Hmgcs2* (encoding HMG-CoA Synthase 2) were increased in the hearts of pancreatectomised rats (Fig. 5B,C; Px effect of *p* < 0.0001 for both targets). As such, *Hmgcl* expression was 1.4-fold higher in the hearts of pancreatectomised rats compared with sham-operated controls, whilst expression of *Hmgcs2* was 14.2-fold higher in the same hearts.

In contrast, expression of *Bdh1* (encoding β-hydroxybutyrate dehydrogenase) and *Oxct1* (encoding succinyl-CoA:3-ketoacid CoA transferase) were suppressed in the hearts of pancreatectomised rats compared with sham-operated rats (Fig. 5D,E; Px effect of *p* < 0.0001 for both targets). Expression of *Bdh1* was 74% lower in the hearts of vehicle-administered, pancreatectomised rats and 83% lower in those treated with isoprenaline, whilst *Oxct1* expression was respectively 58% and 60% lower in the same hearts.

### Myocardial high-energy phosphate and cyclic nucleotide levels

**Figure 6:**
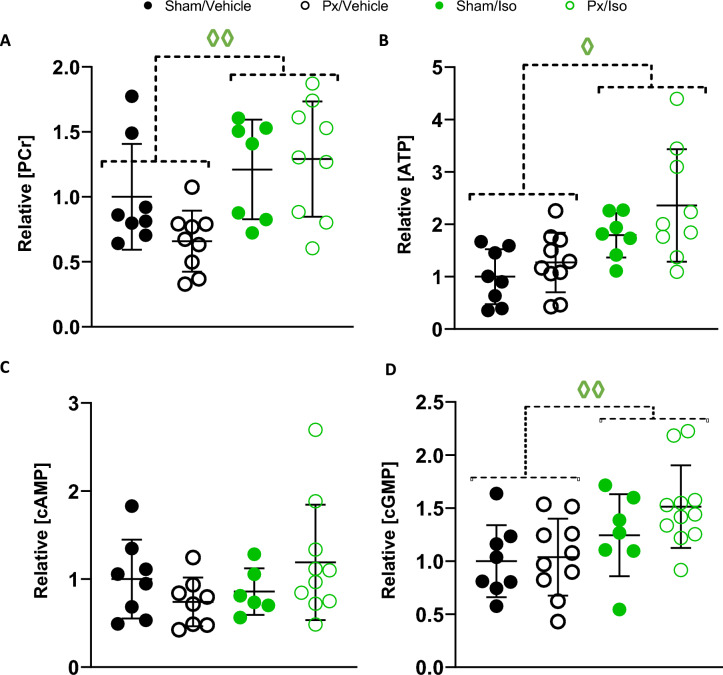
Myocardial high-energy phosphates and cyclic nucleotides. Concentration of (**A**) Phosphocreatine (PCr), (**B**) ATP, (**C**) cAMP and (**D**) cGMP relative to levels in the Sham/Vehicle hearts. All displayed as mean ± SD. 2-Way ANOVA ^◊^*p* < 0.05 and ^◊◊^*p* < 0.01 effect of Iso. n = 7–10 for all groups.

We next considered phosphocreatine (PCr) and ATP levels in the hearts of pancreatectomised and isoprenaline treated rats. Px/Vehicle rats exhibited 35% lower myocardial PCr levels than Sham/Vehicle rats (Fig. 6A), though no difference in ATP levels (Fig. 6B). PCr levels in Px/Iso rat hearts were not lower than in Sham/Vehicle rats however (Fig. 6A; Iso effect, *p* < 0.01), and had 2.4-fold higher levels of ATP (Fig. 6B, Iso effect, *p* < 0.05. Isoprenaline administration thereby appeared to increase high energy phosphate availability; a finding that aligned with the protection of mitochondrial respiratory capacity in the hearts of these rats.

Following isoprenaline administration, levels of AMP and ADP levels were 15.0% and 31.0% higher respectively in Sham/Iso rat LV vs Sham/Vehicle (Supplementary Fig. 2A,B, Iso effects of *p* < 0.05 and *p* < 0.01 respectively). Creatine levels, meanwhile, were 30.9% greater in Px/Vehicle LV than in Sham/Vehicle LV (Supplementary Fig. 2C, Px effect, *p* < 0.01).

Finally, to understand the mechanistic basis for our findings and considering our previous data showing that pancreatectomy altered the myocardial expression of genes encoding mediators of adrenergic signalling and cyclic nucleotide metabolism^4^, we sought to understand the impact of pancreatectomy and isoprenaline on myocardial levels of cAMP and cGMP. We found that neither pancreatectomy nor isoprenaline had a significant effect on myocardial cAMP levels (Fig. 6C), however isoprenaline increased cGMP levels in rat heart (Fig. 6D; Iso effect of p < 0.01).

## Discussion

In this study, we sought to understand the metabolic consequences of β-adrenergic stimulation, via repeated isoprenaline administration, on cardiac substrate metabolism in the 90% pancreatectomy rat model of type 1 diabetes. We found that pancreatectomy resulted in a suppression of cardiac mitochondrial respiratory capacity, but a relative preservation of fatty acid oxidation capacity and altered expression of genes associated with ketone body metabolism. Isoprenaline administration also suppressed mitochondrial respiratory capacity in the hearts of sham-operated rats, but in contrast preserved oxidative phosphorylation capacity in the hearts of pancreatectomised rats. Moreover, isoprenaline altered the expression of genes associated with glucose metabolism alongside levels of myocardial glycolytic intermediates, and increased myocardial cGMP levels.

A strength of this study was the use of the 90% pancreatectomy rat model, which recapitulates many of the features of type 1 diabetes including hyperglycaemia, hypoinsulinaemia and poor glucose tolerance following a reduction in β-cell mass. This model of type 1 diabetes holds advantages over other models for the study of diabetic cardiomyopathy, in that the direct toxic effects associated with streptozotocin administration on tissues including the heart^2,25–27^ are avoided, along with the confounding effects of obesity and/or altered leptin signalling seen in models such as the ZDF rat. Limitations of this surgical model, however, include the abrogation of pancreatic endocrine and exocrine functions beyond effects on insulin secretion. In particular, the impact of pancreatectomy on digestive enzyme secretion could include poor nutrient absorption, weight loss and malnutrition, whilst loss of endocrine glucagon signalling, including cardioprotective effects^28^ and effects on tissue metabolism^29^, may also be a feature of this model.

A further strength of this study was the detailed metabolic phenotyping we carried out at the tissue level, including high-resolution respirometry for the analysis of mitochondrial respiratory capacity under different substrate-led pathways, alongside targeted metabolite analysis via mass spectrometry and metabolic gene expression. Whilst respirometry, carried out ex vivo in the presence of saturating concentrations of substrates and oxygen, reflects capacity rather than metabolic flux in vivo, there was agreement between these measurements and those derived from other techniques. For instance, the relative preservation of mitochondrial fatty acid oxidation capacity seen in permeabilised cardiac fibres from pancreatectomised rats was reflected in the elevated expression of genes encoding enzymes of β-oxidation in these same hearts. Respirometry in permeabilised fibres does not however allow for the measurement of glycolytic flux, which we have instead investigated by measuring concentrations of glycolytic intermediates, and the expression of glycolytic enzymes by LC–MS and RNAseq. We do, however, acknowledge that a limitation of our study is the lack of true measurements of metabolic flux, which could for example be made using stable isotopes, and future studies might investigate some of the pathways we highlight here in a more targeted manner using this approach.

In previous work we reported that isoprenaline did not exacerbate the pathological remodeling associated with type 1 diabetes in the hearts of 90% pancreatectomised rats. In line with this, pancreatectomised rats were protected against isoprenaline-induced mortality in comparison with their sham-operated counterparts^4^. As seen in this study and previous work^18–20^, isoprenaline administration has been recorded to induce mortality, albeit often at higher doses. The mechanism for this is not known, however the findings of this study may shed some light on the mechanism, since pancreatectomy was found to abrogate the effect. This protection may have arisen as a consequence of the downregulation of ADRB2 (encoding the β2-adrenoreceptor) in these rats^4^, decreasing myocardial sensitivity to adrenergic stimulation, in line with previous findings in the diabetic heart^21^. Indeed, isoprenaline was found to induce a weaker inotropic response in type 1 diabetic rat hearts than in non-diabetic hearts, both in vivo and ex vivo^30,31^. Isoprenaline stimulation of the heart has been reported to be augmented by enhanced pancreatic secretion of glucagon^32^, itself an inotropic agent, and as such abrogated glucagon production may be another mechanism of protection in pancreatectomised rats.

The metabolic consequences of the two interventions employed here differed greatly and did not appear to be exacerbated when isoprenaline administration was superimposed upon pancreatectomised rats. Pancreatectomy was associated with a relative protection of mitochondrial fatty acid oxidation capacity, in the face of overall suppression of mitochondrial respiratory capacity, and enhanced expression of genes supporting fatty acid oxidation. These findings correspond with reports in other rodent models of diabetes^33,34^, in particular STZ-induced models of type 1 diabetes^35^, with the mechanism likely to involve stimulation of the fatty acid-activated transcription factor, peroxisome proliferator-activated receptor α (PPARα)^14^. In addition, pancreatectomy resulted in the altered expression of genes involved in ketone body metabolism, including a marked upregulation of *Hmgcs2*, which encodes the canonically-ketogenic enzyme HMGCS2, and downregulation of *Oxct*, which encodes the ketolytic enzyme SCOT. Increased expression of HMGCS2 was previously reported in the hearts of STZ-induced type 1 diabetic rat hearts^36^, and this may have consequences for the susceptibility of the diabetic heart to ischaemia–reperfusion injury^37^.

In contrast with the effects of pancreatectomy, repeated isoprenaline administration did not specifically alter the myocardial expression of genes associated with fatty acid oxidation or ketone body metabolism; however it did result in an overall suppression of mitochondrial respiratory capacity, in conjunction with our previous report of suppression of left ventricular ejection fraction in this model^4^. This finding corresponds to a previous report describing the suppression of both fatty acid oxidation and markers of mitochondrial content to a similar degree in the rat heart following chronic treatment with isoprenaline via osmotic minipumps^20^. Similar observations of suppressed oxidative metabolism have been made in the chronically-infarcted rat heart, which further exhibited lower rates of glycolysis^38,39^. In the present study, we did not find evidence of an overall suppression of glucose metabolism following isoprenaline administration, but instead found increased expression of *Aldoc* (encoding aldolase C), *Pdk1* and *Pdk2*, alongside enhanced levels of glycolytic intermediates downstream of aldolase. We did not see differences in left ventricular lactate concentrations, however lactate can be rapidly exported from myocardium, and we did not measure lactate efflux. Isoprenaline did increase cardiac levels of high energy phosphates. The mechanism behind this isoprenaline-mediated increase in ATP is unknown, however levels of AMP and ADP, alongside the later metabolites in the pentose phosphate pathway, were also raised, hinting that increased levels of purine synthesis may play a role. These findings may collectively be suggestive of altered glycolysis in the face of suppressed oxidative metabolism, but this would need to be confirmed by direct measurements of glycolytic flux.

Of note, both pancreatectomy and isoprenaline administration independently suppressed overall mitochondrial respiratory capacity in the rat heart, but when isoprenaline was superimposed upon pancreatectomy, mitochondrial respiratory capacity was preserved. The mechanistic basis for this finding may again lie in the contrasting effects of the two interventions on adrenergic signaling, with pancreatectomy resulting in desensitisation to adrenergic overload following sustained isoprenaline administration, but isoprenaline stimulation offsetting the accompanying metabolic suppression of type 1 diabetes to some degree through enhanced myocardial cGMP levels. Indeed, cGMP is a mediator of mitochondrial biogenesis in mammalian cells and tissues, acting at least in part through PPARγ co-activator 1α (PGC1α)^40^. Augmentation of cGMP signaling has been proposed as a therapeutic strategy for heart failure with preserved ejection fraction (HFpEF)^41^, and this might be achieved through phosphodiesterase (PDE) inhibition^42,43^ or administration of nitrate/NO-mimetics^44,45^. Moreover, increased myocardial cGMP signalling has been proposed as a possible mechanism underlying the therapeutic benefits of SGLT2 inhibition^46^, and this was associated with enhanced fatty acid oxidation and overall ATP production in the hearts of type 2 diabetic (*db/db*) mice^47^.

Our findings shed new light on the interaction between diabetes-associated myocardial alterations and other factors driving cardiac pathology, underlining the importance of investigating such features in combination. Of note, an investigation that superimposed pressure overload (via transverse aortic constriction, TAC) onto *db/db* mice revealed that the diabetic mice were relatively protected against the functional consequences of TAC in comparison with non-diabetic counterparts^3^. More strikingly, whilst TAC resulted in energetic and metabolic perturbations in the hearts of non-diabetic mice, it led to the restoration of cardiac glucose uptake and high-energy phosphate levels in the hearts of *db/db* mice^3^, inviting the question of whether two wrongs can make a right when it comes to metabolic arithmetic^48^.

Our work has implications for the future use of the model we have deployed here and for possible therapeutic avenues targeting metabolism in the diabetic heart. Previously we sought to investigate the potential of combining pancreatectomy with isoprenaline to establish a novel “dual-hit” model of diabetic cardiomyopathy^4^. Taken together, the findings we described previously and here reveal differing structural, functional and metabolic effects associated with the two stressors, and no exacerbation of the pathology with the combined insult. Moreover, the protection of mitochondrial respiratory capacity in the hearts of isoprenaline-treated pancreatectomised rats, alongside the prevention of isoprenaline-induced mortality following pancreatectomy, have given us cause to re-evaluate this model. Conversely, our work lends support to therapeutic strategies aiming to allay the metabolic/mitochondrial derangements in the diabetic heart through the elevation of cGMP, via routes that may include NO/nitrate supplementation or GLP-1/glucagon signaling.

## Conclusion

The induction of type-1 diabetes via pancreatectomy and chronic β-adrenergic stimulation through isoprenaline have contrasting effects on myocardial substrate metabolism. Moreover, whilst both stressors suppress at least some aspects of cardiac mitochondrial respiratory capacity, when administered in combination respiratory capacity is protected. Our work underlines the importance of studying such stressors in combination when modeling cardiac pathology in rodents, and in particular when considering downstream metabolic consequences.

### Supplementary Information

Supplementary Figures.

## Data Availability

Data from this study is available upon publication at the University of Cambridge Online Data Repository using the following link: 10.17863/CAM.107569. The corresponding author may be contacted to request data from the study.
