# Supplementary material for: Beta-adrenergic agonism protects mitochondrial metabolism in the pancreatectomised rat heart
Source: Sci Rep. 2024 Aug 21;14:19383. doi: 10.1038/s41598-024-70335-4 (PMC11339431; doi:10.1038/s41598-024-70335-4)
Supplement: Supplementary file 1 — Supplementary Figures. [file 41598_2024_70335_MOESM1_ESM.docx]

**Supplementary Figures: Beta-adrenergic agonism protects mitochondrial metabolism in the pancreatectomised rat heart**

Ross T. Lindsay^1,2,3,4^, Louise Thisted^2^, Nora E. Zois^2,5^, Sebastian T. Thrane^2^, James A. West^3,6^, Keld Fosgerau^2,7^, Julian L. Griffin^3,8^, Lisbeth N. Fink^2,9^, Andrew J. Murray^1^

^1^Department of Physiology, Development and Neuroscience, University of Cambridge, UK

^2^Gubra A/S, Hørsholm Kongevej 11b, 2970, Hørsholm, Denmark

^3^Department of Biochemistry and Systems Biology Centre, University of Cambridge, UK

^4^Current Address: Rejuveron AG, Cambridge, United Kingdom

^5^Current Address: Ascendis Pharma A/S, Hellerup, Denmark

^6^Current Address: AstraZeneca, Cambridge, UK

^7^Current Address: Pephexia Therapeutics ApS, Copenhagen, Denmark

^8^Current Address: The Rowett Institute, University of Aberdeen, UK

^9^Current Address: Ferring Pharmaceuticals A/S, Kastrup, Denmark

For Correspondence: Dr Ross T. Lindsay

Rejuveron, Cambridge, United Kingdom.

ross@rejuveron.com


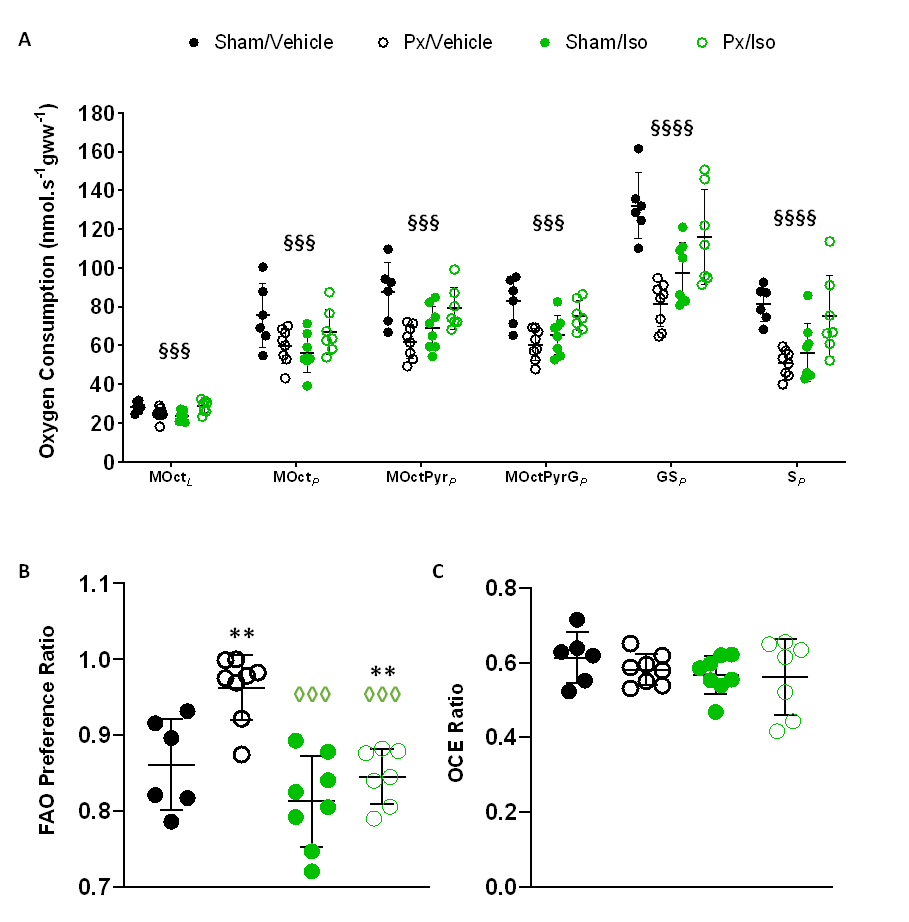
Supplementary Figure 1

**C**

**A**

*****

**D**

**B**

*****

**Supplementary Figure 1:** **Pentose Phosphate Pathway Intermediates and Lactate.** Left-ventricular **(A)** 6-phosphoglycerate, **(B)** Ribulose-5-phosphate, **(C)** Ribose-5-phosphate, and D) Lactate concentrations relative to sham/vehicle, as measured by LC-MS. n = 7-11 for all groups. All results displayed as mean ± SD. 2-way ANOVA effects of Px and Iso: * = *p* < 0.05.


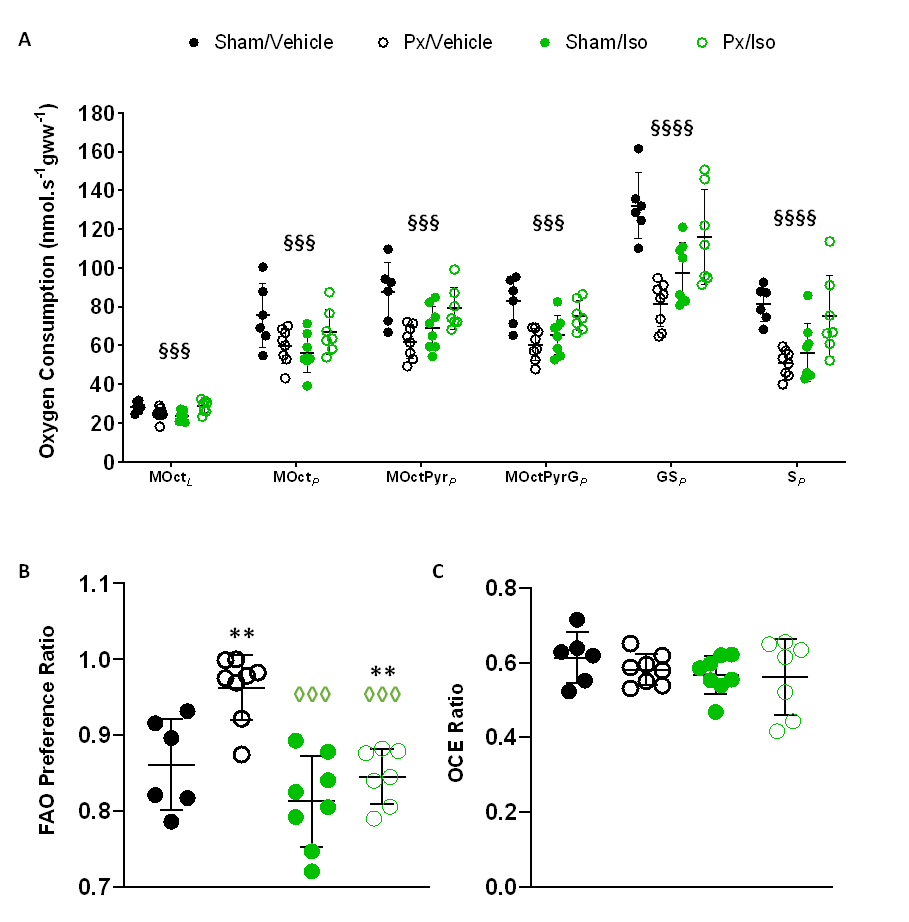
Supplementary Figure 2

******

**A**

******

*****

**B**

**C**

**Supplementary Figure 2:** Left-ventricular **(A)** AMP, **(B)** ADP, and **(C)** Creatine (Cr) concentrations relative to sham/vehicle, as measured by LC-MS. n = 7-11 for all groups. All results displayed as mean ± SD. 2-way ANOVA effects of Px and Iso: * = *p* < 0.05, and ** = *p* < 0.01.
